# Software Usability Testing Using EEG-Based Emotion Detection and Deep Learning

**DOI:** 10.3390/s23115147

**Published:** 2023-05-28

**Authors:** Sofien Gannouni, Kais Belwafi, Arwa Aledaily, Hatim Aboalsamh, Abdelfettah Belghith

**Affiliations:** 1 Department of Computer Science, College of Computer and Information Sciences, King Saud University, Riyadh 11543, Saudi Arabia; kais.belwafi@ku.ac.ae (K.B.); a.aledaily@gmail.com (A.A.); hatim@ksu.edu.sa (H.A.);; 2 C2PS Center, Electrical Engineering and Computer Science Department, Khalifa University, Abu Dhabi P.O. Box 127788, United Arab Emirates

**Keywords:** usability testing, emotion detection, Brain-Computer Interface, channel selection, EEG signal processing, deep-learning, recurrent neural network

## Abstract

It is becoming increasingly attractive to detect human emotions using electroencephalography (EEG) brain signals. EEG is a reliable and cost-effective technology used to measure brain activities. This paper proposes an original framework for usability testing based on emotion detection using EEG signals, which can significantly affect software production and user satisfaction. This approach can provide an in-depth understanding of user satisfaction accurately and precisely, making it a valuable tool in software development. The proposed framework includes a recurrent neural network algorithm as a classifier, a feature extraction algorithm based on event-related desynchronization and event-related synchronization analysis, and a new method for selecting EEG sources adaptively for emotion recognition. The framework results are promising, achieving 92.13%, 92.67%, and 92.24% for the valence–arousal–dominance dimensions, respectively.

## 1. Introduction

Usability testing is a crucial aspect of software development that aims to test software’s ease of use and improve the design and development processes [1]. It is a non-functional software testing requirement, as defined in ISO 9241. It covers three main topics: effectiveness, which consists of testing the achievement of the system functional goals and measuring the accuracy of the system; efficiency, which focuses on resource consumption; and satisfaction, which measures the user experience [2]. Another important aspect of software testing is reliability testing. This is a complement of the above measurements, and involves evaluating the reliability of the system features and, at the same time, determining whether they meet the user’s satisfaction.

User experience (UX) assesses the internal state of the user when using a system by examining how the features, design, functions, complexity, and other aspects of the system affect the mental state of the user while using the system [3]. UX consists of several elements, as stated in [4]. One of the main components of UX is emotions. Several methods have been developed to study emotions in the context of usability testing. However, it is important to acknowledge that traditional approaches, which heavily rely on surveys, technical interviews, and questionnaires, have certain limitations when it comes to accurately distinguishing between different emotional states. These conventional methods often rely on subjective self-report measures, which are susceptible to biases, recall errors, and varying interpretations. Recognizing the shortcomings of these traditional approaches, researchers have sought alternative methods that can provide a more objective and reliable assessment of emotional states. One such promising approach is the utilization of facial expressions, speech, text, or body posture [5,6], and electroencephalography (EEG) signals [3,7]. EEG is a reliable and cost-effective technology used to measure brain activities associated with different emotional responses. By capturing the electrical activity of the brain, EEG provides valuable insights into the neural processes underlying emotions.

Two main approaches have been developed to analyze and classify emotions. The first approach assumes that there is a specific brain behavior and pattern for every emotion. Conversely, the second approach allows for the study of emotions based on broader aspects. It defines several dimensions to create an effective framework for studying emotions instead of studying groups of discrete emotions. There is no limitation to the number of dimensions. However, there are three dimensions used in most previous studies. This is called the valence–arousal–dominance (VAD) model. The dimension valence reflects whether the emotional state correspond to a pleasant state or not. The dimension arousal determines the degree of excitement during the emotion. The dimension dominance represents how much the excitement is controlled.

Few studies have analyzed emotions in usability testing using EEG signals. EEG signals correspond to involuntary brain activity, which reflects the real mental state of a person [8]. The analysis of EEG signals may help in identifying the emotional state in every testing time period with a high accuracy rate [8,9]. For example, in [10], the researchers classified emotions as positive or negative during usability testing. The study sought to determine whether a low-quality user interface could stimulate negative emotions and evaluate whether brain activity analysis could be used to detect the level of usability. The researchers embedded usability tasks in web pages and used the results of a self-assessment questionnaire. The system was validated on 21 participants, after which discrete wavelet transform (DWT) was applied to extract alpha and beta signals from the recorded EEG signals. The DWT algorithm classified emotions with an accuracy of 85.6%.

Another study undertaken in [9] showed promising results when the difficulty level associated with using an interface was analyzed based on brain activity. The researchers classified a user’s perceptions from ‘difficult’ to ‘easy’ when using the Facebook interface. In the study, the features were extracted from 13 electrodes fixed on the scalp and positioned across various regions to capture brain activity. In turn, linear discriminant analysis (LDA) and a support vector machine (SVM) were used to classify the difficulty level. The accuracy was 63% for LDA and 65% for the SVM.

Another study examined in the same context the usability of the Facebook interface with emotion recognition [11]. The researchers built an automatic emotion recognition system, and its results were compared to those of self-assessment manikin data regarding the Facebook interface. The study involved the use of 16 electrodes situated in different brain regions. The researchers applied independent component analysis (ICA) on EEG sources, and power spectrum density (PSD) features were used to calculate valence and arousal values. Finally, the researchers compared the results from the SAM data and the mapped emotional state from valence and arousal. The results indicate that non-Facebook users experienced different emotional states compared to Facebook users [11].

This paper introduces an original framework for usability testing by leveraging electroencephalography (EEG) brain signals for emotion detection. With EEG being a reliable and cost-effective technology for measuring brain activities, this framework holds great potential for impacting software production and enhancing user satisfaction [12]. The proposed approach offers a comprehensive understanding of user satisfaction with high accuracy and precision, making it a valuable tool in software development. The framework incorporates a recurrent neural network algorithm as a classifier, a feature extraction algorithm based on event-related desynchronization and event-related synchronization analysis, and a novel method for the adaptive selection of EEG sources for emotion recognition.

The remainder of this paper is organized as follows. Section 2 introduces the methodology and describes the proposed framework and the signal processing chain. Section 3 presents the experimental results and the evaluation criteria of the proposed system. Section 4 discusses the obtained results and two experimental approaches applied during the user experience using emotions. Finally, Section 5 provides concluding remarks and the possible future research directions on usability testing based on emotion detection using the EEG signals.

## 2. Methods

**Figure 1:**
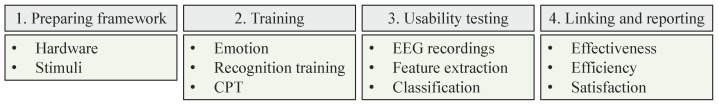
Proposed framework for usability testing with emotion recognition [12].

A generic framework is proposed for the assessment of software usability based on the brain–computer interface (BCI). Figure 1 presents the proposed framework for usability testing with emotion recognition. It relies on detecting a user’s emotions while performing tasks related to a given software system in serial sessions. The emotions are detected and classified in each session using the valence–arousal–dominance (VAD) model. Valence and arousal levels can be used as indicators to predict a user’s satisfaction during their experience with a system. These levels can indicate whether a system causes boredom, stress, anger, or relief, as well as many other emotional states [13]. Building this framework involves the following phases as described in Figure 1: preparation, training, testing, and reporting the usability aspects and linking them with the recognized emotions.

### 2.1. Preparation

The proposed framework is an interface of different choices of web-based systems. EEG acquisition hardware is required to record brain activity in the training phase and for usability testing. Many acquisition systems exist in the market, such as GreenTek [14], OpenBCI [15], Gtech [16], to name a few. Such devices produce high-quality signals, are easy to set up, and are easy to use. The acquired EEG signals are processed, extracted, and then classified using deep learning algorithms. The second factor involved in the experiment is the emotion stimuli. The emotional stimulus in this work was the DEAP dataset [17]. This is consistent with the proposed method; 40 videos were used in the experiment under the same setting.

### 2.2. Training Phase

The training phase of this proposed algorithm consists of two parts: emotion recognition training and continuous performance test (CPT).

#### 2.2.1. Emotion Recognition Training

This phase aims to train the proposed algorithm on the brain activities of the subjects who are enrolled in the experiment. The videos used in the chosen dataset were applied for the subjects in this phase under the appropriate setting. In this stage, either the locationist or VAD models can be applied. In turn, a self-assessment questionnaire can be used to specify the emotion of the participant (either discrete status or valence–arousal–dominance status). In the last one, we can follow the same setting as that described in [17]. Furthermore, valence and arousal levels can be specified using the validated formulas given in [18]. The latter criteria should be studied more with the proposed method because they involve the selection of specific electrodes from specific frequency bands [18]. However, they are applicable.

#### 2.2.2. Continuous Performance Test

Continuous performance test (CPT) method assists in specifying the response time for each subject, which reflects the first impression period. It is an important factor for recognizing the emotion that arises in the first impression period, and so it plays an essential role in studying the satisfaction of each user during usability testing [19]. CPT measures the response of clicking the ‘space’ key when any character appears on the screen, except for the ‘X’ character. The response time is the first impression time for a given user [19].

### 2.3. Usability Testing Phase

In this phase, the user chooses a website to test its usability. The procedure is divided into multiple ‘to-do’ tasks. Each task has a time where it begins and a time where it ends, and the user attempts to complete the task in the defined period. This phase can be divided into two implicit sessions:

1. *First-impression testing*: As we previously measured CPT for each subject, this period is analyzed later to determine the user’s first impression of the website at the beginning, as well as for each task.
2. The rest of the time is the EEG recording to facilitate emotion recognition for a particular task.

#### 2.3.1. EEG Signal Processing Chain

##### Pre-Processing EEG Signals

In order to achieve accurate results from EEG data, it is crucial to utilize precise processing techniques and noise removal methods. In the DEAP dataset [17], the electrooculogram (EOG) data that produce artifacts resulting from eye movements were removed. To extract the alpha, beta, theta, and gamma signals for each trial, a fast Fourier transform (FFT) filter was implemented in conjunction with a common average referencing (CAR) filter and a high-pass filter. EEGLAB toolbox was used to perform CAR and FFT processing. EEGLAB is an interactive MATLAB toolbox designed for processing and filtering EEG and MEG signals [20].

##### Feature Extraction

This is the main concept in emotion recognition. The quality of extracting features from EEG signals underpins the accurate recognition of emotions. The brain activities were acquired using EEG electrodes placed on the scalp, following the international 10–20 system. Although scalp EEG recordings do not provide precise spatial information about specific brain regions, it is essential to acknowledge the inter-variability that exists between subjects and emotions. Effective brain activities associated with different emotions can vary not only between different emotions but also among individuals. In this respect, sensitive brain regions for each subject were distinguished [21]. In turn, we selected the EEG channels with high activity compared to the neutral status of the person individually. Following this, the channels were selected from the significant regions in order to ensure that an adaptive channel selection method was used. Finally, the feature vector was constructed from the values of the ERD/ERS of the chosen electrodes (or from the difference in PSD between asymmetrical electrodes). In fact, each chosen electrode has an activity in at least one emotional state. ERD/ERS measures the EEG power for a subject in a certain band for a group of trials (related to an emotion), as well as its power as a percentage relative to the power of the trial of a baseline (neutral in this case) of the same band and subject. In this part, we calculated the ERD/ERS values of all the significant electrodes for all eight emotional states, as given in Equation (1):

(1) $$ERD/ERS=\frac{\left(A-R\right)}{R},$$

where *A* is the active average power and *R* is a reference average power. The reference is the state that must be compared to the brain activity. It depends on the study applied [22]. More details about the feature extraction method can be found in [21,23].

##### Classification

**Figure 2:**
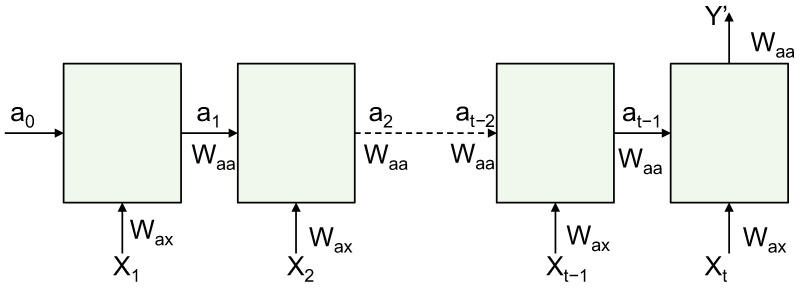
Architecture of a recurrent neural network.

There are two types of final emotion labels: VAD model labels and distinct labels of emotional state, as represented by the locationist model. In turn, the results were evaluated to determine which classifier method outperformed the others. Therefore, this section focuses on improving the method using a recurrent neural network (RNN) model. An RNN is a deep learning neural network that processes sequential data on a time axis. It is an improvement of the convolutional neural network (CNN), which is limited by a fixed number of inputs and outputs, as well as by the fixed flow of data in the hidden layer. In an RNN, the number of inputs and outputs is flexible (e.g., one-to-one, one-to-many, and many-to-many). In addition, RNNs can process data in a loop by passing information from a layer to the same layer, and information can be memorized from a previous layer. As a result, RNNs are suitable for sequential and dependent data, which has yielded valuable applications in text recognition, speech recognition, conversion of a rating to text (and back again), and sentiment analysis. The structure of an RNN involves a neural network, which consists of an input layer, hidden layers, and an output layer. The input and output can be a sequence of data. Each RNN layer is a combination of a number of hidden layers with the same weights and biases (Figure 2).

For each timestep *t*, the activation $a_{t}$ and the output $y_{t}$ are expressed as follows:

(2) $$a_{t}=g\left(W_{aa}a_{t-1}+W_{ax}X_{t}+b\right),$$

and

(3) $$y_{t}=g\left(W_{ya}a_{t-1}+b\right)$$

where $a_{t}$ is the current state of the RNN current layer, $a_{t-1}$ is the previous state of the previous layer, and *X* is the input for that RNN layer. Additionally, $W_{aa}$ and $W_{ax}$ are weight vectors, and *b* is the bias factor. Furthermore, *g* represents the activation function, which is given as ReLU, sigmoid, or hyperbolic tangent. The activation function for this work is as follows:

(4) g(t)=tanh(t)

An RNN can be used with different error minimization methods, especially for avoiding the vanishing gradient problem. In this research, the Levenberg–Marquardt (LM) algorithm was used, which solves the non-linear least squares problem [24]. It combines two methods used in error minimization between function points, namely the gradient descent method and the Gauss–Newton algorithm. The LM reduces squared errors by changing the parameters with the steepest-descent direction, which is the same as in the gradient descent method. If the values are near the optimal value, the parameters of the squared error are changed by assuming the function is quadratic and then finding the value of the local quadratic, as in the Gauss–Newton algorithm [24]. The aim of LM is to find the best perturbation *h* to parameter *p* for minimizing the error value represented by $X^{2}$ in this formula:

(5) $$\begin{array}{c} X^{2}\left(p\right)=\sum_{i=1}^{m}{\left[\frac{y\left(t_{i}\right)-\hat{y}\left(t_{i};p\right)}{\sigma_{yi}}\right]}^{2}={\left(y-\hat{y}\left(p\right)\right)}^{T}W\left(y-\hat{y}\left(p\right)\right)\\ =y^{T}Wy-2y^{T}W\hat{y}+{\hat{y}}^{T}W\hat{y} \end{array}$$

where $X^{2}$ is the sum of the weighted square errors of the main $y(t_{i})$ data and the curve fit function $\hat{y}\left(t_{i};p\right)$. $\sigma_{yi}$ is the measurement error of the measurement $y(t_{i})$ and, finally, *W* is the weight matrix, calculated by $W_{ii}=\frac{1}{\sigma_{yi}^{2}}$. As mentioned above, LM seeks to find the best perturbation *h* to parameter *p*, as shown in the following:

(6) $$\begin{array}{c} \left[J^{T}WJ+\lambda I\right]h_{lm}=J^{T}W\left(y-\hat{y}\right) \end{array}$$

where *J* comes from

(7) $$\hat{y}\left(p+h\right)≅\hat{y}\left(p\right)+\frac{\partial \hat{y}}{\partial p}h=\hat{y}+Jh$$

The λ increases or decreases depending on the approximation of $X^{2}\left(p+h_{lm}\right)⩾X^{2}\left(p\right)$ in each iteration. The small values of the damping parameter λ result in a Gauss–Newton update and large values of λ result in a gradient descent update.

**Figure 3:**
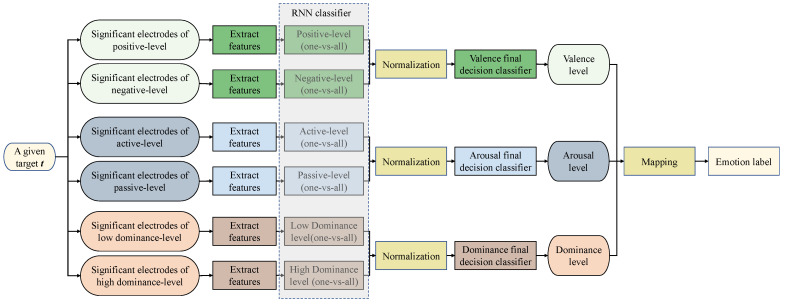
Classification of emotions based on the VAD model using an RNN classifier.

1. **Classification Scheme Using RNN**: Emotion classification on EEG data from the DEAP dataset [17] was performed using an RNN classifier. The classification process proceeded through the following two levels:
  - ‘One-vs.-all’ level: A trial *t* is input into two classifiers from type ‘one-vs.-all’. The first is a high-level classifier (positive in valence, active in arousal, and low in dominance). The second classifier is a low-level classifier (negative in valence, passive in arousal, and high in dominance). The feature vector for each classifier includes the event-related desynchronization (ERD) and event-related synchronization (ERS) from the significant electrodes of the corresponding level. Figure 3 illustrates the structure of the classification process.
  - ‘Final-decision classifier’ level: For each dimension, there is a final decision classifier that decides on the level of the dimension from the two ‘one-vs.-all’ classifiers related to that dimension, as shown in Figure 3. The criteria used to inform the final decision are given in Table 1.
2. **Neural Network Structure:** The considered classifier is based on an RNN with the LM algorithm, Jacobian matrix calculation, and real-time recurrent learning. It uses gradient descent to find the optimal values of weights, as well as a bias factor that gives a low cost and error factor. The general structure of an RNN is illustrated in Figure 2. However, details were obtained through various experiments, as well as research surrounding the issue of what constitutes an optimal structure. Finally, the highest results in terms of accuracy were obtained when the structure configuration is as follows:
  - The network consists of several input layers, equal in number to the total number of selected significant electrodes. The size of the feature vector is different from subject to subject, depending on the subject’s brain activity.
  - There are 80 hidden layers for receiving each feature vector component. Each layer is an MLP layer with a loop inside, along with delay factors. The MLP layers contain 80 neurons for receiving input components, and then the activation function *tanh* is applied.
  - The proposed classification system is nearly static. In particular, the delay between inputs is set to be a naive value in order to avoid focusing on dependencies in emotions between trials. The DEAP dataset used independent videos in the experiment. The aim to set naive values for delay is to take advantage of RNN in training the data.

### 2.4. Linking and Reporting

This is the final phase in the proposed framework. The emotions were linked to the usability testing aspects—effectiveness, efficiency, and satisfaction—and a report was generated.

## 3. Results

### 3.1. Overview of the DEAP Dataset

**Figure 4:**
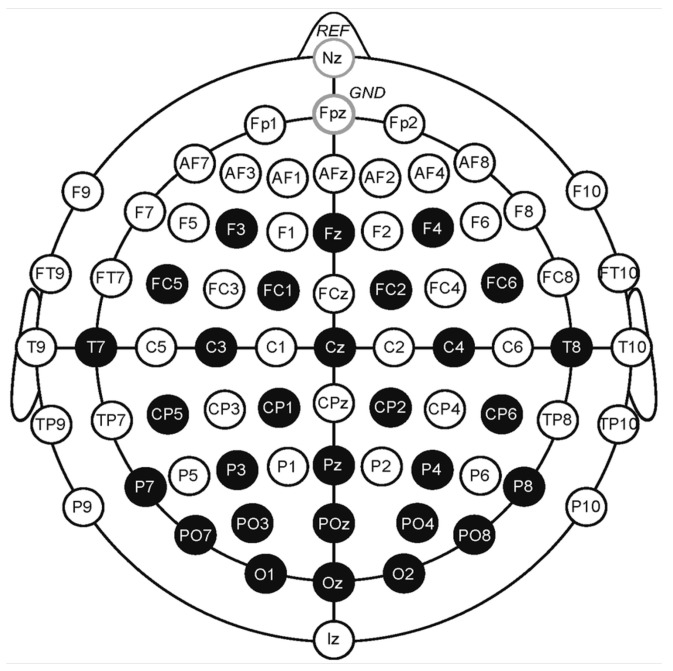
Initial electrodes used in this research as the DEAP dataset.

One of the most well-known datasets in the area of emotion identification from EEG signals is the DEAP dataset [17]. A total of 32 participants’ emotional states were monitored as they watched music videos. Each subject was recorded over the course of 40 movies (40 trials), which were captured at a sampling rate of 512 Hz before being down-sampled to 128 Hz utilizing 40 channels, of which 32 were EEG electrodes. Figure 4 presents the spatial partition of the electrodes to the related brain regions. Each color refers to a brain region: frontal, central, temporal, occipital, and parietal lobe.

Arousal, valence, dominance, and liking/disliking were the four labels assigned to each video/trial for each subject. Using a self-assessment questionnaire, the participants also scored the valence, arousal, dominance, and like/dislike. Frontal face footage was also taken for 22 of the 32 participants. Retrieval by affective tags from the database was employed as a novel technique for selecting stimuli.

The number of trials conducted for each subject at a particular level of a specific dimension was insufficient. Thus, each trial was divided into equal-sized fragments. Consider a trial *t* that lasts for *T* units of time, with each fragment having a window size of *W* units of time. The overlapping ratio between two consecutive fragments is denoted by O, which determines the amount of shared data. Trial *t* was divided into *N* fragments of size *W*, with *N* calculated using the following equation (Equation (8)):

(8) $$\begin{array}{c} N=(\frac{\left(T-w\right)}{shift})+1\\ shift=(1-O)W \end{array}$$

For our experiment, we selected a window size of 12 s for each fragment and an overlapping ratio of 50%. As a result, each trial *t* was split into eight fragments, effectively increasing the number of trials by eight.

### 3.2. RNN Configuration Settings

Different values and settings were tested in order to find the optimal performance and accuracy for the RNN classifier. Table 2 lists the settings of the RNN used in the simulation of the work.

### 3.3. Training and Testing Data

The processed data were augmented with a window of size 12 s and an overlap ratio of 50%. As proposed, the network’s input size was set to *n*, which represents the number of significant electrodes. The proposed method was implemented using the MATLAB pyrenn toolbox [25], as well as the MATLAB machine learning toolbox.

### 3.4. Performance Evaluation

As previously mentioned, the first stage involved each dimension having two one-class classifiers to distinguish between the high and low levels. The second stage involved making a final decision between the one-class classifiers in each dimension. Table 3 shows the results of using an RNN with the VAD model to estimate emotions during the usability testing of a software on 32 subjects. The model was able to estimate three dimensions of emotion—valence, arousal, and dominance—at two levels each: positive/negative, active/passive, and low/high, respectively.

The model achieved an overall accuracy of above 90%, which is quite good [26]. Specifically, for valence, the model achieved an accuracy of 90.19% for positive valence and 92.13% for negative valence. This means that the model is better at identifying negative valence than positive valence, which could indicate that the software being tested is more likely to elicit negative emotions.

For arousal, the model achieved an accuracy of 92.13% for active arousal and 92.67% for passive arousal. The model performed slightly better at identifying passive arousal, indicating that the software may be more likely to elicit passive emotions.

Finally, for dominance, the model achieved an accuracy of 92.24% for low dominance and 91.78% for high dominance. This means that the model is better at identifying low dominance than high dominance, which could indicate that the software being tested is more likely to make users feel less in control.

Overall, the results suggest that the RNN with the VAD model is effective at estimating emotions during software usability testing, and could provide valuable insights into how users feel about the software.

Using the RNN, the accuracy increased by 3% in valence, 8% in arousal, and 15% in dominance compared to the previous results in [21]. Additionally, each level classifier exceeded 90% in terms of accurate emotion recognition. The confusion matrices of the final decision classifier for each dimension are presented in Table 4(a–c).

**Figure 5:**
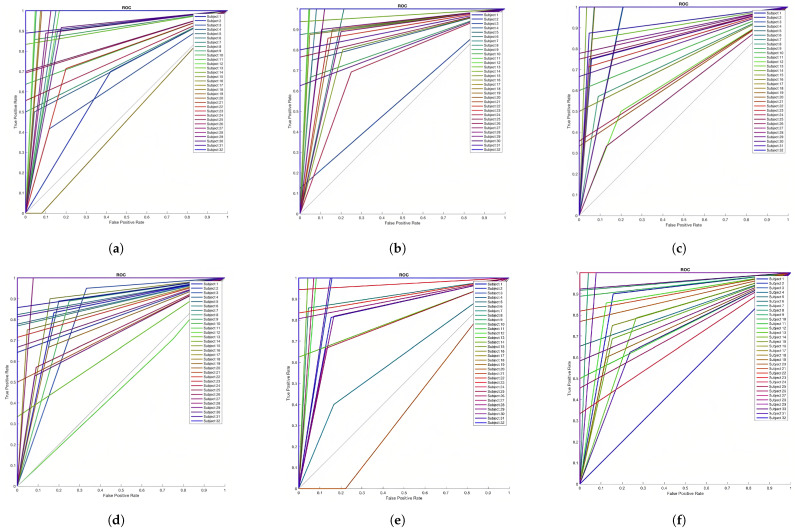
ROC curves for all dimensions in classification process using RNN. (**a**) Valence—negative level; (**b**) Valence—positive level; (**c**) Arousal—passive level; (**d**) Arousal—active level; (**e**) Dominance—low control level; (**f**) Dominance—high control level.

The receiver operating characteristic (ROC) curves for all subjects are presented in Figure 5 for the three dimensions. The ROC represents the level of separability; in other words, how much this classifier is capable of distinguishing between true positive and false positive. From Figure 5, we can conclude that the model, in all dimensions, has an acceptable separability feature for 96–100% of the subjects.

## 4. Discussion

The performance of the proposed system was comparatively examined against the results yielded by other recent methods in the field (see Table 5). All of the methods used as a basis for the comparison had similar characteristics, including the fact that they used the VAD model for emotion description, the DEAP dataset, and a deep learning technique in the classification stage.

The results attest to the value of the RNN when applied to the proposed VAD-based emotion recognition system. The extracted features from an adaptive-channel selection algorithm were used to train an RNN classifier. The results indicate that the RNN outperformed the quadratic classifier with VAD dimensions. Moreover, comparing the results of the RNN method to recent studies reveals that the proposed system outperformed other state-of-the-art approaches in the field of DL applied to emotion recognition.

**Figure 6:**
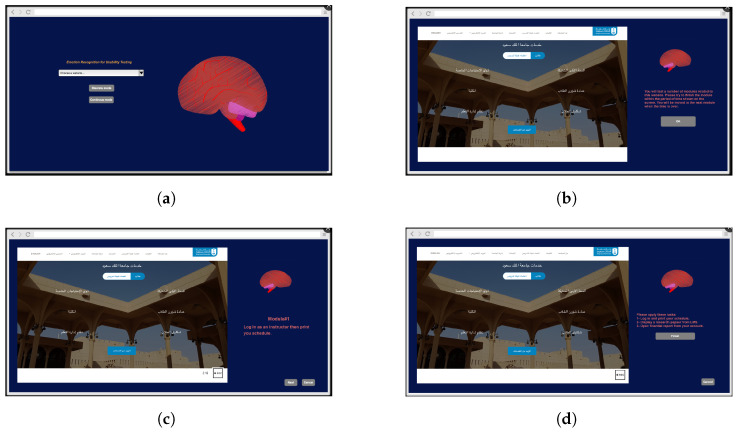
Example of testing framework. (**a**) Main screen of the prototype framework. (**b**) Instruction screen. (**c**) Module testing screen. (**d**) Continuous mode in usability testing experiment.

Two experimental approaches are applied when studying the user experience using emotions [13], which are discrete and continuous approaches. The first one involves applying specific events and analyzing physiological responses over a short-term period. The continuous approach involves studying physiological states over a long-term period under different circumstances. Arousal and valence levels are measured in this period. It is crucial to establish a research goal before undertaking an experiment. In this work, we applied both approaches. In the main screen of the interface, the user can choose between the two testing approaches, as shown in Figure 6a.

### 4.1. Discrete Method

The user opens the website and starts to complete the tasks shown on the list (see Figure 6c). Each module has a start time and an end time, and the user must complete the task within the defined period; otherwise, the next module begins.

**Figure 7:**
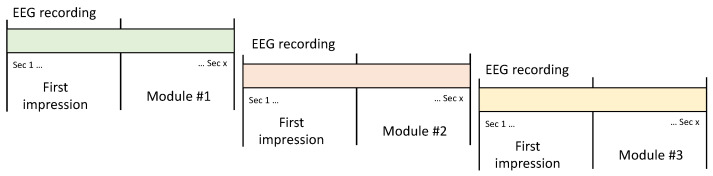
EEG recording segments in discrete mode.

The EEG recording is separate for each module, as shown in Figure 7.

### 4.2. Continuous Method

EEG recordings are continuous from the start of the session to the end, and no timer is established for each module. The user ends each module and moves on to the next one while the EEG is recorded, as in Figure 7.

Later, the recording is divided depending on the module’s task periods. The first impression period is specified in the beginning only in this method (Figure 6d).

Linking and reporting is the final phase in the proposed framework. The emotions were linked to the usability testing aspects—effectiveness, efficiency, and satisfaction—and a report was generated. Related to usability testing factors, we linked the proposed framework processes with these factors in the following way:

- *First impression factor*: The classification result of the EEG period in CPT allows the user’s first impression to be determined. This helps to improve design aspects such as the use of color, alignment, typography, and so on.
- *Task-based testing factor*: This is mapped with the classification results of each module. The feature vector is constructed for each module recording. The emotion label of the classifier is the subject’s emotional state during this task. This factor helps in improving the task design, and it has a direct impact on usability.
- *Overall emotions factor*: This factor is shown in the continuous method of the framework. It is an important factor for facilitating a measurement of usability when we divide the recording of each module and the satisfaction in general if we take the results of the entire continuous period as a feature vector.

Furthermore, the approach to testing is linked to all aspects of usability testing, as shown in the following:

- *Effectiveness*: In the ‘task-based test’, the level of each dimension in VAD is an indicator of the quality of the design and the function of the interface [13]. The correlation measurement is an accurate indicator of the emotion and the task/function of the system during the user experience, which reflects effectiveness [32].
- *Efficiency*: Based on the definition of efficiency given in [2], groups of emotions (e.g., calm or miserable) are linked to measurements of resource consumption. The type and level of correlation measurement leads to the level of system efficiency [2].
- *Satisfaction*: The appearance of emotions that are either pleasant or unpleasant during the testing session can be used to draw an inference about user satisfaction. It is an indicator of each task, function, and the overall system. Scenarios such as the first impression test and free interaction test [33] are chosen in this step. Certain measurements are suggested at this point to decide on the satisfaction level, including the percentage change relative to a baseline state (i.e., neutral emotion or calm), correlation, and the means of the changes [2,33].

Regarding the VAD model, the dominance dimension has not been explored in any depth in the context of usability testing [34]. Furthermore, there are no significant correlations in usability testing [35] or in acceptance technology in general [36]. This aspect should be examined and, in particular, researchers should seek to uncover new facts about the relationship between dominance and usability testing. Another open question relates to the issue of how to map this dimension to usability testing factors. However, this does not omit the valuable role of dominance in determining an individual’s emotional state during usability testing.

## 5. Conclusions

This paper presents a comprehensive study on the use of deep learning (DL) techniques for emotion recognition using the VAD model. The main focus of the study was to evaluate the performance of the recurrent neural network (RNN) in determining the levels of each dimension of the VAD model for emotion recognition.

The results of the study show that the proposed RNN method outperforms other methods, including a quadratic classifier, in recognizing emotions based on VAD dimensions. It surpasses the performance of state-of-the-art approaches in DL-based emotion recognition.

In addition to the evaluation of the emotion recognition system, the paper also outlined an original usability testing framework that leverages the developed emotion recognition system. The framework aims to provide objective, reliable, and valid measurements of usability testing linked to a user’s emotional states. It is designed with two modes, discrete and continuous, to cater to well-defined goals in usability testing.

By utilizing the accuracy of the proposed emotion classification system, the paper’s usability testing framework serves as a foundation for an efficient, reliable, and automatic usability testing process. Overall, the paper provides valuable insights into the use of DL techniques for emotion recognition and a viable foundation for an efficient, reliable, and automatic usability testing framework.

## Figures and Tables

**Table 1:** Criteria majority voting classification.

Final Decision Criteria		Classifier of Upper Level	
		(Target) Label	(Outlier) Label
Classifier of Lower Level	(target) label	Error	Lower level of the dimension
	(outlier) label	Upper level of the dimension	Neutral level of the dimension

**Table 2:** RNN settings in emotion recognition with VAD model.

Factor	Value
Adapt damping factor of LM	10
Damping factor of LM	3
Delay factor	0.01
Input nodes	Same as the number of adaptive significant electrodes
Hidden layers	80
Nodes in each hidden layer	80
Output layers	1
Weight	Random values [−0.5, +0.5]
Bias	Random values [−0.5, +0.5]
Epochs	8
Error E (Cost function)	$110^{-10}$

**Table 3:** RNN with VAD model results.

Dimension	Level	Accuracy (%)	Precision (%)	Recall (%)	Specificity (%)	TPR(%)	F1-Score (%)	Decision (%)
Valence	Positive	90.19	84.12	94.12	86.24	94.12	88.84	85.88
	Negative	92.13	84.13	94.66	88.72	94.75	89.08	
Arousal	Active	92.13	80.12	95.45	88.33	95.95	87.11	87.32
	Passive	92.67	91.18	90.52	92.80	92.52	90.85	
Dominance	Low	92.24	83.63	96.06	88.72	96.06	89.41	87.56
	High	91.78	88.76	93.31	90.56	93.31	90.98	

**Table 4:** Confusion matrix of final decision classifier.

(a) Valence				
		**Predicted Class**		
**Actual Class**	**Positive**	**Neutral**	**Negative**	**Error**
Positive	81.26 (TP)	14.77	1.10	2.85
Neutral	4.25	91.47 (TN)	2.57	1.69
Negative	2.22	13.64	82.71 (TN)	1.41
**(b) Arousal**				
		**Predicted Class**		
**Actual Class**	**Active**	**Neutral**	**Passive**	**Error**
Active	80.35 (TP)	10.11	5.14	1.26
Neutral	3.28	88.66 (TN)	7.12	0.92
Passive	0	7.53	87.50 (TN)	1.83
**(c) Dominance**				
		**Predicted Class**		
** Actual Class**	**Low Dominance**	**Neutral**	**High Dominance**	**Error**
Low dominance	72.63 (TP)	11.39	3.43	3.16
Neutral	0.62	94.70 (TN)	4.14	0.52
High dominance	1.59	8.24	76.21 (TP)	1.44

**Table 5:** Comparison of proposed method and related works.

Method	Number of Classes	Feature Extraction Algorithm	Number of Electrodes	Classifier	Valence (%)	Arousal (%)	Dominance (%)
[27]	2 levels of valence and arousal	EMD and MEMD	18	ANN	75	72.87	N/A
[28]	2 levels of valence and arousal	Time- and frequency- domain, with DE as selection method	5	PNN	67.47	67.47	N/A
[29]	2 levels of valence and arousal	EEG raw signals	32	RNN	85.65	85.45	N/A
[30]	2 levels of valence and arousal	PSD and VMD	4	DNN	62.50	61.25	N/A
[31]	2 levels of valence and arousal	HOS	32	Proposed NN	85.21	84.16	N/A
Proposed method	2 levels of valence, arousal, and dominance	ERD/ERS of the significant electrodes	Adaptive	RNN	85.88	87.71	86.63

## Data Availability

Not applicable.
